# Atlanta Hospital

**Published:** 1881-01

**Authors:** 


### Editorial.

### ATLANTA HOSPITAL.

THE very commendable and charitable efforts that have recently been inaugurated to establish a permanent and efficient City Hospital where sick strangers and destitute invalids may have the benefit of good nursing and proper medical attention, we are happy to see, have been crowned to some extent with the success they so richly deserve.

There is no doubt in the minds of those who have the good of the city at heart that the establishment of such an institution is almost essential to our municipal prosperity. This need has long been felt, and, but for the proverbial lack of unanimity among members of the medical profession, there would probably, long since, have been established an institution that the city would have been proud of. Professional jealousy and bickering has continually, however, thwarted so desirable a consummation, and it has devolved at last upon the charitable, self-sacrificing Sisters of Mercy, by untiring and assiduous exertions, to establish a Hospital that is worthy the support and patronage of every good citizen. With an eye single to the good of the destitute and helpless sick, these good Sisters have struggled on through the worst vicissitudes of weather and against the opposition of many selfish and contrary individuals, until they have caused to be established an Atlanta Hospital, where they may be found night and day uobly and disinterestedly ministering to the comforts of the sick under their charge.

Who can deny the benefit of this enterprise, or who could withhold support and assistance from so commendable an effort?

It has been demonstrated to be an utter impossibility for a hospital to be established under the auspices of the medical profession. The city could not well move in such an enterprise without professional concurrence; at the same time there can be no doubt as to the necessity of such an institution in Atlanta. In this emergency assistance has been produced, and from most appropriate sources. No better or more devoted attendants upon the sick can be found than this noble band of women who willingly devote their time and sacrifice their comforts in the discharge of their self-imposed duties at the Hospital. It should be the sacred duty of every member of the profession to sustain them, since they are one and all interested alike, and there can be no occasion of the envy and jealousy that has heretofore crippled every similar undertaking.

Atlanta is far behind in hygienic improvements. It is high time that definite systems should be established looking to the continuation of the salubrity we now enjoy. The rudimentary arrangements now existing may be well enough at present, in our comparative infancy, but with increased population will come *pari passu* increase of deleterious accumulations and other unhealthy conditions, to meet which our present systems will be found utterly inadequate. Improvements in drainage and in facilities for removing unhealthy accumulations, a remodeling of our present system of water works and in a substantial addition in the way of funds and official as well as individual support to our Hospital are some of the most urgent requirements that the city should turn her attention to.

